# Prediction of nucleosome rotational positioning in yeast and human genomes based on sequence-dependent DNA anisotropy

**DOI:** 10.1186/1471-2105-15-313

**Published:** 2014-09-22

**Authors:** Feng Cui, Linlin Chen, Peter R LoVerso, Victor B Zhurkin

**Affiliations:** Thomas H. Gosnell School of Life Sciences, Rochester Institute of Technology, Rochester, NY 14623 USA; School of Mathematical Sciences, Rochester Institute of Technology, Rochester, NY 14623 USA; Laboratory of Cell Biology, National Cancer Institute, NIH Bg. 37, Room 3035A, Convent Dr., Bethesda, MD 20892 USA

**Keywords:** Nucleosomes, Rotational positioning, Sequence-dependent DNA anisotropy, Prediction of nucleosome positioning

## Abstract

**Background:**

An organism’s DNA sequence is one of the key factors guiding the positioning of nucleosomes within a cell’s nucleus. Sequence-dependent bending anisotropy dictates how DNA is wrapped around a histone octamer. One of the best established sequence patterns consistent with this anisotropy is the periodic occurrence of AT-containing dinucleotides (WW) and GC-containing dinucleotides (SS) in the nucleosomal locations where DNA is bent in the minor and major grooves, respectively. Although this simple pattern has been observed in nucleosomes across eukaryotic genomes, its use for prediction of nucleosome positioning was not systematically tested.

**Results:**

We present a simple computational model, termed the W/S scheme, implementing this pattern, without using any training data. This model accurately predicts the rotational positioning of nucleosomes both *in vitro* and *in vivo*, in yeast and human genomes. About 65 – 75% of the experimentally observed nucleosome positions are predicted with the precision of one to two base pairs. The program is freely available at http://people.rit.edu/fxcsbi/WS_scheme/. We also introduce a simple and efficient way to compare the performance of different models predicting the rotational positioning of nucleosomes.

**Conclusions:**

This paper presents the W/S scheme to achieve accurate prediction of rotational positioning of nucleosomes, solely based on the sequence-dependent anisotropic bending of nucleosomal DNA. This method successfully captures DNA features critical for the rotational positioning of nucleosomes, and can be further improved by incorporating additional terms related to the translational positioning of nucleosomes in a species-specific manner.

**Electronic supplementary material:**

The online version of this article (doi:10.1186/1471-2105-15-313) contains supplementary material, which is available to authorized users.

## Background

Nucleosomes play a critical role in gene regulation in eukaryotes by modulating the access of various transcription factors to DNA [1]. Genome-wide data on *in vivo* nucleosome organization in yeast reveal that nucleosomes are depleted in the promoter regions [2], providing space for assembly of the transcriptional machinery. Accurate determination of nucleosome positions is extremely important when studying gene regulatory mechanisms because displacement of a nucleosome by just a few nucleotides may occlude (or expose) the binding site of a protein. Nucleosome positioning is usually characterized by two parameters: rotational positioning, referring to the side of the DNA helix that faces the histones, and translational positioning, determining the nucleosome midpoint (or dyad) with regard to the DNA sequence [3]. Various experimental and computational methods have been proposed to provide high-resolution mapping of nucleosomes (see below).

The most commonly used empirical method for nucleosome mapping involves treating native chromatin with micrococcal nuclease (MNase), which has been employed to generate genome-wide nucleosome maps in many eukaryotes [4–8]. However, it is well documented that MNase has strong sequence preferences: it cuts predominantly within AT-rich sequences in both free DNA [9, 10] and in the linker DNA between nucleosomes [11, 12]. This sequence specificity makes it difficult to determine the boundaries of nucleosomes bordered by GC-rich sequences [13].

The free hydroxyl radical (FHR) method was originally used to study the structure of DNA and DNA-protein complexes [14]. It has several advantages over MNase cleavage. First, hydroxyl radical footprinting has no pronounced sequence preference [15]. (At the same time, the extent of hydroxyl radical cleavage can be used to obtain information on sequence-dependent variation in DNA shape [16].) Second, the small size of FHRs in solution allows them to cut the DNA backbone at every nucleotide that is not protected by protein(s). Later, Flaus *et al*. [17] developed the site-directed hydroxyl radical (SDHR) approach to precisely map nucleosome dyads. Using this approach, researchers have successfully determined 16 nucleosome positions *in vitro* at a single base-pair resolution [17–24] (see Table 1). Recently, this approach was used to map *in vivo* nucleosome positions across the yeast genome [25]. These precise experimental nucleosome positions serve as ideal test cases for computational approaches to nucleosome positioning prediction.

**Table 1:** **Rotational positioning of** ***in vitro*** **nucleosomes predicted by the two computational schemes**

Number	Nucleosome positioning fragment	Exp. dyad position	Exp. method	W/S scheme	KS-2009 model	Reference
1	'601'	134	SDHR/FHR	+	+	[18, 19]
2	'603'	153	FHR	+	+	[19]
3	'605'	131	FHR	+	+	[19]
4	*X. borealis* somatic 5S rDNA	−24	SDHR	+	–	[20]
5	"	−3	SDHR	+	+	[20]
6	"	+7	SDHR	+	+	[20]
7	"	+48	SDHR	+	+	[20]
8	"	+58	SDHR	+	–	[20]
9	*X. borealis* oocyte 5S rDNA	−2	SDHR	+	+	[20]
10	"	+20	SDHR	+	+	[20]
11	"	+34	SDHR	–	+	[20]
12	"	+58	SDHR	+	–	[20]
13	Sea urchin 5S rDNA	−11/–12	SDHR	+	+	[17]
14	"	+8	SDHR	+	–	[17]
15	MMTV	−127	SDHR	+	+	[22]
16	"	+70	SDHR	+	+	[22]
17	pGUB	84	SDHR	–	–	[23]
18	"	104	SDHR	–	–	[23]
19	Fragment 67	113	FHR	–	+	[24]
20	Chicken β^A^-globin	−281	MNase + DNase I	–	–	[21]
			Total correct predictions	15/20	13/20	

FHR: free hydroxyl radical method; SDHR: site-directed hydroxyl radical method. The experimental dyad positions and methods used are given according to the references.

The extent of agreement between the experimental data and the predictions: the symbol ‘+’ indicates that the discrepancy between the predicted maximal score and experimental dyad position does not exceed 2 bp. The symbol ‘–’ indicates that the predicted and experimental positions are separated by 3–6 bp (that is, about a half helical turn of DNA duplex).

Computational models for nucleosome positioning can be roughly divided into two classes: structure-based models and sequence-based models. The structure-based models are based on analyses of structural parameters of individual dinucleotide steps derived from crystal structures of nucleosome core particles and numerous protein-DNA complexes [26]. Nucleosomal DNA is severely deformed when wrapped around the histone octamer. Several models have been proposed to assess the energy cost of the deformations required to wrap DNA around the histone core [19, 27–31] and to calculate the DNA structural features [32] which can be used for prediction of the nucleosome occupancy and transcription factor binding [33].

The sequence-based models depend on statistical analyses of sequence features in nucleosomal DNA fragments. It has been known for many years that certain sequence motifs usually occur at particular sites within a nucleosome, constituting characteristic patterns. The initial breakthrough was made by Trifonov and Sussman [34], who observed periodic oscillations of dinucleotides, especially AA:TT, in genomic sequences and postulated that they are critical for bending of DNA and stabilization of nucleosomes. Since then, various features have been suggested to be essential for DNA packaging in chromatin [35]. The most well-known sequence pattern is related to the rotational setting of nucleosomes. That is, AT-containing dinucleotides (AA, TT, AT and TA, denoted as WW) frequently occur in the minor-groove sites facing toward the histone, while GC-containing dinucleotides (GG, CC, GC and CG, denoted as SS) are often found in the minor-groove sites facing outward. This pattern has been observed in nucleosomal DNA from chickens [36], yeast [4, 8], fruit flies [6], nematodes [5] and humans [7], indicating that the structural rules for rotational positioning are essentially the same across species.

The WW, SS and other similar patterns were extensively used for prediction of the nucleosome positioning. In particular, Ioshikhes and colleagues analyzed the correlation profiles for the AA/TT and GG/CC dinucleotide patterns [6, 37, 38]. Reynolds *et al*. [39] compared mono-, di- and tri-nucleotides and found that the mono-nucleotide patterns are the most informative features. Tillo and Hughes found that G + C content dominates nucleosome occupancy [40], while Chung and Vingron further showed that the overall G + C preference for nucleosomal DNA together with the periodic dinucleotide patterns results in maximal predictive performance [41]. Teif and Rippe used the aforementioned DNA patterns, as well as remodeler activities to predict nucleosome positions [42].

At the same time, other research groups used large nucleosome occupancy data sets to develop discriminative models [43, 44] and regression-based models [45, 46], which aim to predict nucleosome positions at low resolution by discriminating between nucleosome and linker DNA. These studies show that genome-wide nucleosome occupancy is often directed by exclusion signals such as long A-tracts.

The Segal group initially developed a Markov model incorporating the aforementioned periodic patterns associated with nucleosome rotational positioning and taking into account steric exclusion and thermodynamic equilibria [8]. This model was later modified by introducing a “position-independent” component, *P*_L_, to represent sequences that are generally favored or disfavored regardless of their position within the nucleosome (most notably, poly(dA:dT) tracts, which are strongly disfavored by nucleosomes) [11, 47]. This method, denoted as KS-2009 hereafter, is quite successful in predicting *in vivo* nucleosome occupancy across the yeast genome [47]. The notation KS-2009 gives credit to the first and the last authors of the paper (Kaplan and Segal).

Note that the term “position” has two different meanings in the above description – the first is the *position* of a nucleosome on DNA, and the second is a *position* along the nucleosome length. To avoid possible confusion, the second case will be denoted as a “site” on nucleosomal DNA. Accordingly, the above value *P*_L_ will be denoted below as a “site-independent” component. (This component can also be described as a “translational component,” as it distinguishes between the sequences favorable for nucleosome cores and for linkers – see below).

Recently, we developed a method (denoted as the YR scheme) aiming to predict the exact positioning of nucleosomes *in vitro* [48]. It was based on analysis of the periodic distribution of dinucleotides WW, SS and YR, as well as of the YYRR and RYRY motifs (here Y is pyrimidine and R is purine). The tetranucleotides were included to reflect the differential bending anisotropy of pyrimidine-purine (YR) dinucleotide steps in the context of their neighbors [49, 50]. We found that 17 of the 20 nucleosomes mapped at high resolution *in vitro* are predicted within 2 bp from their experimental positions. Our data showed that both the dinucleotide and the tetranucleotide patterns are critical for nucleosome positioning [48]. However, the relative importance of the WW, SS and YR dinucleotides (as well as of the YYRR and RYRY tetranucleotides) remained unclear.

To address this issue, we used a simple W/S model based solely on distribution of the WW and SS dinucleotides. This model is a modification of the method described earlier [51]. Below, we demonstrate that the W/S model provides accurate prediction of the rotational positioning of nucleosomes both *in vitro* and in the yeast and human genomes, with an error distribution narrower than that produced by the KS-2009 model. We suggest that the W/S model, in conjunction with the translational component *P*_L_ introduced by Kaplan *et al*. [47], has a potential for accurate prediction of both the rotational and translational positioning of nucleosomes *in vivo*.

## Methods

### *In vitro*experimental nucleosome positions

Twenty nucleosome positions were mapped *in vitro* using high-resolution mapping techniques such as the FHR and SDHR methods (see Table 1 and Additional file 1: Table S1 in ref. [48]). All these positions were used in this study.

### *In vivo*experimental nucleosome positions

Three sets of nucleosome positions mapped *in vivo* at high resolution are used in this study. One set is from yeast, mapped by the SDHR method [25], while two other sets, one from yeast and one from humans, are mapped by MNase cleavage [52, 53]. The SDHR Brogaard set [25] includes 67,548 unique nucleosome dyad positions across the yeast genome, 8 of which are too close to the ends of chromosomes (*i.e*., the distances are less than 73 bp.). The remaining 67,540 positions were used in this analysis. The MNase Cole set contains ~5 million fragments from yeast with lengths from 147 to 152 bp [52]. Only fragments 147 bp in length (number = 783,455) were used in this analysis. The MNase Gaffney set contains ~2.5 billion paired-end reads with lengths between 126 and 184 bp from seven human lymphoblastoid cell lines [53]. Only the 147-bp fragments (number = 133,735,124) were used in this study. Note that ~16% of yeast nucleosomes and ~5% of human nucleosomes were selected; our analysis, however, is not exclusively effective with fragments of this length. That is, using nucleosomal DNA fragments with the length L = 145 bp or 149 bp yields similar results.

### W/S scheme

The W/S scheme is based on the method described earlier [51] with some modifications. Briefly, this method implements the well-established sequence patterns initially observed by Travers and his colleagues in chicken nucleosomes [36]. That is, the WW dinucleotides predominantly occur at the sites of DNA bending into the minor groove, while the SS dinucleotides are frequently found at the sites where DNA is bent toward the major groove. In this implementation, the 147-bp and 146-bp nucleosomal templates contain 14 minor-groove bending sites and 12 major-groove bending sites (Additional file 1: Table S1 and Table S2. Additional file 2: Figure S1), each 4 bp in length. (Note that in the earlier version of W/S scheme [51] only 147-bp template was considered).

For example, consider the superhelical location SHL −5.5, which covers the nucleosomal DNA locations 15 through 18 (Additional file 1: Table S1 and Additional file 2: Figure S1). When computing the WW score, C_ww_, for this site, we consider three dinucleotide steps: 15–16, 16–17 and 17–18. If two or three WW dimers occur at this site, C_WW_ = 2 or 3, respectively (*i.e*., if the tetramer 15–18 contains WWW or WWWW motif). This ‘cumulative’ approach is consistent with the idea that three or four consecutive AT pairs are more favorable (compared to a single WW dimer) for interaction with the histone arginines penetrating into the minor groove [28]. Similarly, the WW score is computed for the other DNA-bending sites along nucleosomal DNA. For each 147-bp nucleosomal fragment with the dyad at position *n*, the total score *S*(*n*) is defined as


where C_ww_ and C_ss_ are the total occurrences of WW and SS dinucleotides occurring at a given site. (For brevity, the minor-groove and major-groove bending sites are denoted as minor and major sites, respectively.) That is, the WW fragments occurring at the minor groove sites and the SS fragments occurring at the major groove sites are treated as ‘gains’ because they facilitate anisotropic DNA bending into the minor and major grooves. By contrast, the WW fragments in the major groove sites and the SS fragments in the minor groove sites are considered to be ‘penalties’.

Since both 146-bp and 147-bp DNA fragments can form stable nucleosome core particles [54], it is critical to consider both templates to provide greater flexibility to the model. The profiles for the 147-bp and 146-bp templates were combined in the following way. For a given position *n*, the score of the 147-bp template (spanning the interval from *n*–73 to *n*+73) is compared with the scores of the two 146-bp templates occupying positions from *n*–73 to *n*+72 and from *n*–72 to *n*+73. The locations of the minor- and major-groove sites for both templates are shown in Additional file 1: Tables S1 and S2. The highest of the three scores is assigned to position *n*. The resulting 147/146-bp profile is compared with the experimentally detected nucleosome positions. Note that in our model, the linker DNA is not used for calculation of the W/S score.

### Comparison with other computational models

Our method was compared with a widely used computational model developed by Segal and colleagues, denoted as the KS-2009 model [47]. We used the executable file available at the website (http://genie.weizmann.ac.il/software/nucleo_prediction.html; Version 3 – December 2008). In the output of the KS-2009 model, the “*P* start” values are reported for the probability of a nucleosome starting at a given position. To compare with the W/S score assigned to the center of a nucleosome, we shift the “*P* start” value by 73 bp and denote it as “*P*-center”. In addition, we compared our model with two recent physics-based models, one developed by van der Heijden *et al*., denoted as the HN-2012 model [30], and the other by Minary and Levitt, denoted as the ML-2014 model [31].

## Results and discussion

### Prediction of *in vitro*nucleosome positions mapped at high resolution

**Figure 1:**
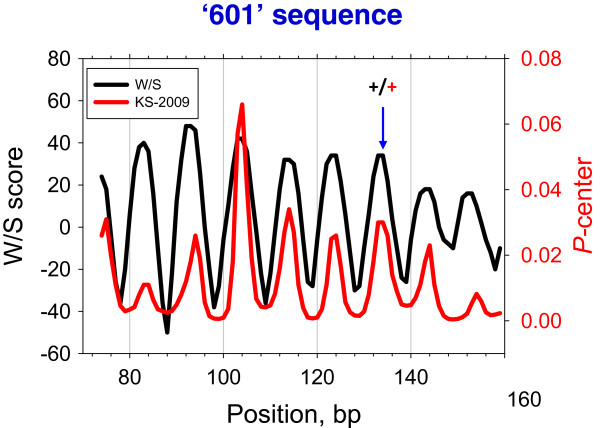
**Prediction of the ‘601’ nucleosome position by the W/S (black) and KS-2009 (red) models (see ‘ Methods ’for details).** The arrow denotes the experimentally determined dyad of the ‘601’ nucleosome. The ‘+’ signs indicate that the predicted positions are within 2 bp from the experimentally determined position.

First, we set out to predict the well-established nucleosome position on the DNA of synthetic clone ‘601.’ It is one of the highest-affinity sequences identified so far for histone binding [55]. Clearly, both the W/S and KS-2009 models fail to predict the translational positioning of the ‘601’ nucleosome because the highest peaks are not at the experimental location (Figure 1). Nevertheless, the two methods do succeed in predicting the rotational positioning of the nucleosome – their profiles show oscillating patterns with a ~10-bp periodicity and have the local maximum at the experimentally determined location. Unfortunately, both the HN-2012 and the ML-2014 models fail to correctly predict the rotational positioning of the ‘601’ nucleosome (Additional file 2: Figure S3 and Additional file 2: Figure S4).

Table 1 summarizes, for each of the 20 experimental *in vitro* nucleosome positions, the predictions made by the W/S and KS-2009 models. Note that most of the 20 positions are mapped by the SDHR method, a very accurate method that can map nucleosome positioning at single base-pair resolution (see Introduction). The W/S scheme correctly predicts the rotational positioning of 15 nucleosomes, but fails in five cases (Figure 1 and Additional file 2: Figure S2). We showed earlier [48] that in additional to the WW and SS dinucleotides, distribution of the tetranucleotides YYRR and RYRY has to be considered to account for positioning of four out of the five nucleosomes mentioned above. This explains why the W/S scheme fails for these nucleosome positions.

The KS-2009 model gives correct predictions for 13 out of 20 positions (Figure 1 and Additional file 2: Figure S2). Notably, the KS-2009 model succeeds in two out of the five positions for which the W/S scheme fails. The most interesting case is the oocyte 5S rDNA fragment [20]. On this fragment, four nucleosomes were mapped at positions −2, +20, +34 and +58 with respect to the transcription start site of the 5S gene. The position +34 is obviously out of phase with the other three positions. The success of the KS-2009 model in predicting the rotational setting of nucleosomes at positions −2, +20 and +34 (Additional file 2: Figure S2H) indicates that this approach, in some cases, can predict nucleosome positions even if they are in the opposite rotational phases. It should be noted, however, that the peaks at positions +20 and +34 are very low compared to the peak at position +48, where no nucleosome was observed experimentally.

**Figure 2:**
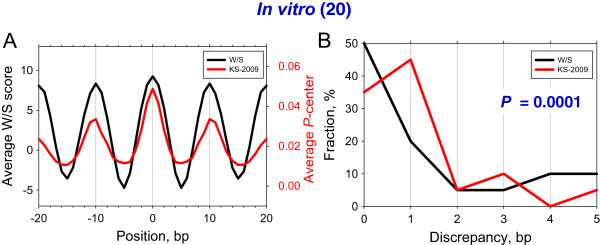
**Predicting 20** ***in vitro*** **nucleosome positions using the W/S and KS-2009 models. (A)** The average W/S score profile (black line) and the average *P*-center profile for the KS-2009 model (red line) [47]. The nucleosomal DNA sequences (Table 1) are aligned around their dyads (position 0); the average score profiles are ‘symmetrized’ with respect to the dyads. **(B)** Error distribution for the two models. The error is calculated as a discrepancy between the experimental position and the position with the highest theoretical score in the interval [−5, +5]. The occurrence of a given error is shown as the percentage of total sequences. For example, in the case of the W/S scheme, 50% of the nucleosome positions were predicted exactly and 20% of the positions were predicted with errors of +/−1 bp. The sum of the fractions is 100%. Here and in Figure 3B, Figure 4B, Figure 4D, the interval [−5, +5] is chosen because the prevalent distance between neighboring nucleosomes is ~10 bp [56, 57].

Taken together, both the W/S and KS-2009 models predict the rotational setting of ~70% of the nucleosomes *in vitro* with the precision of 2 bp (Table 1). This result is based on a detailed case-by-case comparison which is hardly possible for a genome-wide analysis. Therefore, we need to develop an automatic computational procedure for handling millions of nucleosome positions *in vivo*. In an earlier report [51], we made an ‘overall comparison’ of the observed positions with the theoretical score profiles. As follows from Figure 2A, the experimental positions of nucleosomes coincide with the peaks in the averaged predicted profiles. Note, however, that these profiles do not give information about the discrepancy between the experimentally observed and the predicted positions of the nucleosome in each particular case. To quantify how precisely each nucleosome position is predicted, we calculated the error distributions (Figure 2B). Overall, the error distribution for the W/S model differs significantly from the one for the KS-2009 model (*P* = 0.0001 by chi-squared test). The fraction of positions predicted exactly (*i.e*., error = 0) was 50% for the W/S model and 35% for KS-2009 model. Although the fraction of positions with a discrepancy exceeding 2 bp was ~30% for both models (Figure 2B), the W/S model outperformed the KS-2009 model, yielding a narrower error distribution. Importantly, the error distribution gives the same results as the detailed analysis of the 20 nucleosome positions *in vitro* presented above. Thus, we can use this computational approach to evaluate the accuracy of prediction of the nucleosome positioning genome-wide, as manual comparison is impractical.

### Prediction of nucleosome positions in yeast mapped by the SDHR method

**Figure 3:**
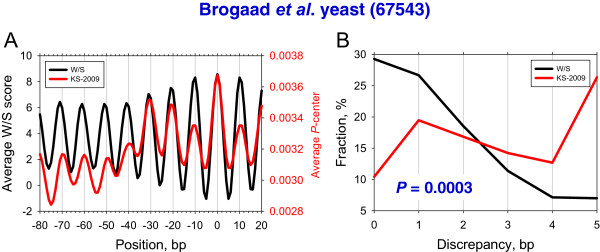
**Prediction of the yeast nucleosome positions mapped by the SDHR method** [25]**, by the W/S and KS-2009 models.** Average score profiles **(A)** and error distributions **(B)** are shown. The notations are the same as in Figure 2.

To compare the performance of the two models in the case of *in vivo* nucleosomes, we first analyzed the yeast nucleosomes mapped by the SDHR method [25]. It is clear that both computational models produce periodic score profiles with maximal values at the experimental dyad positions (Figure 3A). At the same time, the two profiles display noticeable differences in the vicinity of the dyad. In particular, the W/S peak at the dyad (position 0) has almost the same height as the peaks at positions ±10 and ±20, while the KS-2009 peak at the dyad clearly stands out from the rest of the peaks (Figure 3A). Since the KS-2009 model incorporates both periodic dinucleotide patterns (the “site-dependent” component) and the frequencies of penta-nucleotides (the “site-independent” translational component; see above) it is plausible that the observed difference is related to the site-independent part of the model.

A comparison of error distributions for the two models shows that they are significantly different (Figure 3B; *P* = 0.0003 by chi-squared test). For example, the W/S model has the highest fraction of nucleosomes with positions predicted precisely (29%), which is much higher than for the out-of-phase positions with error ±5 bp (~7% of positions). By contrast, the KS-2009 model predicts precisely only ~10% of the nucleosomal positions, while the fraction of the out-of-phase positions increases to ~25%. Moreover, the W/S model predicts ~75% of the *in vivo* positions with the precision of 2 bp, compared to ~45% by the KS-2009 model. These data demonstrate that the W/S model predicts the rotational setting of these nucleosomes fairly well, whereas the KS-2009 model fails to distinguish between the rotational settings of the experimental positions and their immediate neighbors.

### Prediction of yeast and human nucleosome positions mapped by MNase cleavage

**Figure 4:**
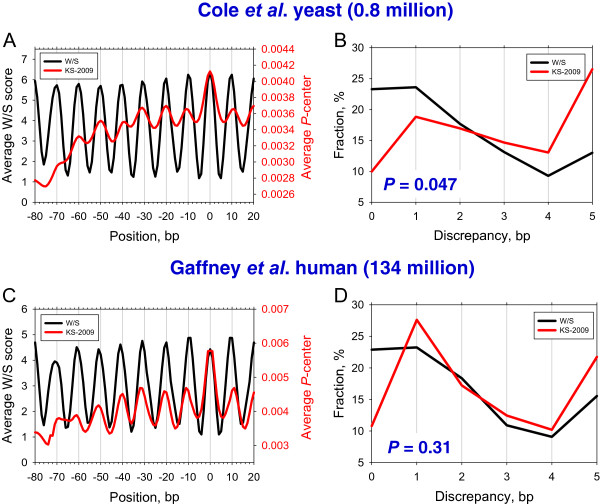
**Prediction of the yeast and human nucleosome positions mapped by MNase cleavage.** Average profiles and error distributions for the W/S and KS-2009 models for yeast **(A**, **B** and human **(C**, **D)** nucleosomes (see Methods). The notations are the same as in Figure 2.

To exclude the possibility that performance of the two models is sensitive to SDHR mapping, we investigated the yeast nucleosomes mapped by MNase cleavage [52]. This dataset was obtained by paired-end sequencing. Thus, the lengths of the nucleosomal DNA fragments were derived precisely. Only 147-bp fragments were used in our analysis (see Methods). As before, the two models produce periodic score profiles with maximal values at the dyad (Figure 4A). Moreover, the profiles produced by the KS-2009 model exhibit the global maxima at the experimental dyad (position 0), consistent with the trend described above (Figure 3A). By analogy with the previous section, the two models yield different error distributions for the MNase set of nucleosomes (see Figure 4B; the two distributions are significantly different, with *P* = 0.047 by chi-squared test). The W/S model predicts ~65% of the nucleosome positions with 2 bp precision, compared to ~45% predicted by the KS-2009 model. Thus, we conclude that the W/S model is better than the KS-2009 model at predicting the rotational nucleosome positioning in yeast, no matter which mapping method (MNase or SDHR) was used.

On the other hand, there is a notable difference between the two yeast sets [25, 52] mapped by different techniques. The W/S score amplitude varies by 10 units for the nucleosomes mapped by the SDHR method [25] (Figure 3A), while it varies by 5 units for the nucleosomes mapped by MNase cleavage [52] (Figure 4A). The SDHR set contains ~70,000 “almost non-overlapping” nucleosome positions selected from a redundant map of ~350,000 nucleosomes [25], while the MNase set contains ~800,000 nucleosome fragments that are 147 bp in length [52], without any additional selection. It is thus possible that the SDHR set is more ‘homogeneous’ due to a specific selection process, which results in a larger variation of the W/S score (between the in-phase and out-of-phase nucleosome positions).

In the case of human nucleosomes, the translational positioning is again predicted better by the KS-2009 model (Figure 4C), while the W/S model performs somewhat better in terms of rotational positioning: it predicts ~65% of the nucleosome positions with 2-bp precision, compared to ~55% for the KS-2009 model (Figure 4D). Accordingly, the difference between the two error distributions is statistically insignificant (*P* = 0.31 by the chi-squared test, Figure 4D). In other words, the W/S and KS-2009 models demonstrate very similar performance when used to map the human nucleosomes.

Finally, note yet another difference between the two models. The W/S model appears to be species-independent – it correctly predicts ~65% of positions for both yeast and human nucleosomes mapped by MNase cleavage (Figure 4B and Figure 4D). By contrast, the KS-2009 model performs differently for the two species – it predicts ~55% and ~45% of positions for the human and yeast nucleosomes, respectively. Ironically, the KS-2009 model was devised based on yeast *in vitro* data [47]. Nevertheless, our analysis indicates that this model performs better for the human nucleosomes mapped *in vivo* [53]. Since chromatin remodeling is involved in nucleosome positioning *in vivo*, the difference in rotational positioning prediction of the KS-2009 model in the cases of yeast and human nucleosomes may reflect different remodeling activities in these two species.

## Conclusions

We have developed the simple and easily reproducible W/S model for prediction of the rotational positioning of nucleosomes based on the well-established sequence-dependent bending anisotropy of DNA [26, 49, 50]. Our model does not use specific training data sets or make any assumptions about the species-dependence of the nucleosome positioning. Therefore it can be used to predict nucleosome positions on any genomic DNA. This, in turn, is important for understanding the molecular mechanisms modulating the access of various transcription factors to DNA in the context of chromatin. For example, recently we used the 147-bp analog of the W/S model to examine accessibility of p53 binding sites in the human genome for the tumor suppressor protein p53 [51]. By contrast, the W/S scheme presented here uses a ‘flexible’ template allowing variation of the nucleosomal DNA fragment from 146 to 147 bp. We know from earlier experience that consideration of the stretching flexibility of DNA is critical for precise prediction of nucleosome positioning, e.g., in the case of the ‘601’ nucleosome [27, 28].

To compare the performance of different models, we used a simple and effective way to evaluate the error distribution. As follows from our study, the W/S scheme is superior at predicting the rotational positioning, whereas the KS-2009 model is more successful in predicting the translational positioning of nucleosomes because it contains a “site-independent” translational component [47].

Naturally, additional training on the high-resolution datasets would improve performance of the ‘sophisticated’ models like KS-2009 containing numerous external parameters. Our main goal, however, was to show that a simple and transparent W/S scheme that was not trained on any data, works ‘reasonably well’ in predicting rotational positioning of nucleosomes. This opens exciting possibility of improving the performance of existing models by combining their ‘positive’ features. It is conceivable that the W/S model might correctly predict the translational positioning of nucleosomes after a species-specific translational component is added.

## Electronic supplementary material

Additional file 1: **Tables S1 and S2 contain description of the minor- and major-groove bending sites in 147-bp and 146-bp nucleosomal DNA fragments.** (DOC 60 KB)

Additional file 2: **This file contains four supplementary figures (Figures S1-S4).** (PDF 253 KB)
