# Supplementary material for: Prediction of nucleosome rotational positioning in yeast and human genomes based on sequence-dependent DNA anisotropy
Source: BMC Bioinformatics. 2014 Sep 22;15(1):313. doi: 10.1186/1471-2105-15-313 (PMC4261538; doi:10.1186/1471-2105-15-313)
Supplement: Supplementary file 1 — Additional file 1: Tables S1 and S2 contain description of the minor- and major-groove bending sites in 147-bp and 146-bp nucleosomal DNA fragments. (DOC 60 KB) [file 12859_2014_6630_MOESM1_ESM.doc]

**Prediction of nucleosome rotational positioning in yeast and human genomes based on sequence-dependent DNA anisotropy**

**Feng Cui, Linlin Chen, Peter R. LoVerso and Victor B. Zhurkin**

**Supplementary Tables**

**Table S1 Minor-groove bending sites in 147-bp and 146-bp nucleosomal DNA fragments^†^**

| **SHL** | **DNA location**  **(147-bp)** | **DNA location**  **(146-bp #1)** | **DNA location**  **(146-bp #2)** |
| --- | --- | --- | --- |
| **-6.5** | [5-8] | [5-8] | [6-9] |
| **-5.5** | [15-18] | [15-18] | [16-19] |
| **-4.5** | [26-29] | [26-29] | [27-30] |
| **-3.5** | [37-40] | [37-40] | [38-41] |
| **-2.5** | [47-50] | [47-50] | [48-51] |
| **-1.5** | [57-60] | [57-60] | [58-61] |
| **-0.5** | [67-70] | [67-70] | [68-71] |
| **+0.5** | [78-81] | [77-80] | [78-81] |
| **+1.5** | [88-91] | [87-90] | [88-91] |
| **+2.5** | [98-101] | [97-100] | [98-101] |
| **+3.5** | [108-111] | [107-110] | [108-111] |
| **+4.5** | [119-122] | [118-121] | [119-122] |
| **+5.5** | [130-133] | [129-132] | [130-133] |
| **+6.5** | [140-143] | [139-142] | [140-143] |

^†^The locations of the minor-groove bending sites are based on the 147-bp template. For the two ‘orientations’ of the 146-template (#1 and #2), the locations are shifted by 1 bp (see Methods).

**Table S2 Major-groove bending sites in 147-bp and 146-bp nucleosomal DNA fragments^†^**

| **SHL** | **DNA location**  **(147-bp)** | **DNA location**  **(146-bp #1)** | **DNA location**  **(146-bp #2)** |
| --- | --- | --- | --- |
| **-6** | [10-13] | [10-13] | [11-14] |
| **-5** | [21-24] | [21-24] | [22-25] |
| **-4** | [32-35] | [32-35] | [33-36] |
| **-3** | [42-45] | [42-45] | [43-46] |
| **-2** | [52-55] | [52-55] | [53-56] |
| **-1** | [62-65] | [62-65] | [63-66] |
| **+1** | [83-86] | [82-85] | [83-86] |
| **+2** | [93-96] | [92-95] | [93-96] |
| **+3** | [103-106] | [102-105] | [103-106] |
| **+4** | [113-116] | [112-115] | [113-116] |
| **+5** | [124-127] | [123-126] | [124-127] |
| **+6** | [135-138] | [134-137] | [135-138] |

^†^The locations of the major-groove bending sites are based on the 147-bp template. For the two ‘orientations’ of the 146-template (#1 and #2), the locations are shifted by 1 bp (see Methods).
