# Supplementary material for: Prediction of nucleosome rotational positioning in yeast and human genomes based on sequence-dependent DNA anisotropy
Source: BMC Bioinformatics. 2014 Sep 22;15(1):313. doi: 10.1186/1471-2105-15-313 (PMC4261538; doi:10.1186/1471-2105-15-313)
Supplement: Supplementary file 2 — Additional file 2: This file contains four supplementary figures (Figures S1-S4). (PDF 253 KB) [file 12859_2014_6630_MOESM2_ESM.pdf]

Prediction of nucleosome rotational positioning in yeast and human genomes based on sequence-dependent DNA anisotropy

Feng Cui, Linlin Chen, Peter R. LoVerso and Victor B. Zhurkin

Supplementary Figures

Figure S1. Locations of the minor- and major-groove bending sites in nucleosomes. DNA fragments with minor grooves facing the histone octamer (*i.e.*, minor-groove bending sites, denoted by "m") are colored in blue. The fragments with major grooves facing the octamer (*i.e.*, major-groove bending sites, denoted by "M") are colored in red. The minor- and major-groove bending sites are represented by their superhelical locations (SHLs) of nucleosomal DNA (–0.5, –1, –1.5,…, –6.5). The 'anterior' half of the nucleosome structure (PDB: 1KX5) is shown. In the 'posterior' half, the minor- and major-groove bending sites are located symmetrically with respect to the dyad (position 0, denoted by a diamond).

Figure S2. Prediction of the nucleosome positions *in vitro* by the W/S and KS-2009 [47] models. The nucleosome positions in the 603 (**A**), 605 (**B**), pGUB (**C**), Fragment 67 (**D**), Sea urchin 5S rDNA (**E**), chicken β-globin gene (**F**), *X. borealis* somatic rDNA gene (**G**), *X. borealis* oocyte rDNA gene (**H**) and MMTV (**I**, **J**) sequences were used for analysis. Symbols are the same as in Figure 1.

Figure S3. Prediction of the '601' nucleosome position by the W/S model (black) and the ML-2014 [31] model (blue). The experimental position is shown by arrow. The W/S score (black) and E_n - E_l values (blue) of the ML-2014 model are presented as a function of the position along the '601' sequence [55]. Note that the full length of the '601' sequence (232 bp long) is used for calculation. For the ML-2014 model, we downloaded the software package Methodologies for Optimization and Sampling in Computational Studies (MOSAICS) (http://csb.stanford.edu/~minary/MOSAICS.html) and associated scripts. We followed the instructions provided by the authors (http://www.cs.ox.ac.uk/mosaics/nucleosome/nucleosome.html) to set up the software. In the output of the ML-2014 model, the "E_n - E_l" values are reported for the nucleosome forming energy of a given 147-bp DNA fragment (where E_n is the energy of the particular sequence on DNA that is bent to fit the nucleosome and E_l is the energy of the same sequence on ideally straight B-DNA, termed "linear DNA").

Figure S4. Prediction of the '601' nucleosome by the W/S model and the HN-2012 model [30]. The W/S scores are compared with the "Free energy, kT" values (**A**), the "Diad" (*a.k.a.* "dyad") values (**B**) and the "Occupancy" values (**C**) as a function of the position along the '601' sequence [55]. For the HN-2012 model (http://bio.physics.leidenuniv.nl/~noort/cgi-bin/nup3_st.py) the calculations were made with the parameters B = 0.2, p = 10.1 bp and N = 74 bp, as specified in the legend to Figure 2 [30]. Note that we failed to reproduce the results presented by van der Heijden *et al.* in their Figure 2 [30]. Arrows indicate the experimentally determined position of the '601' nucleosome.

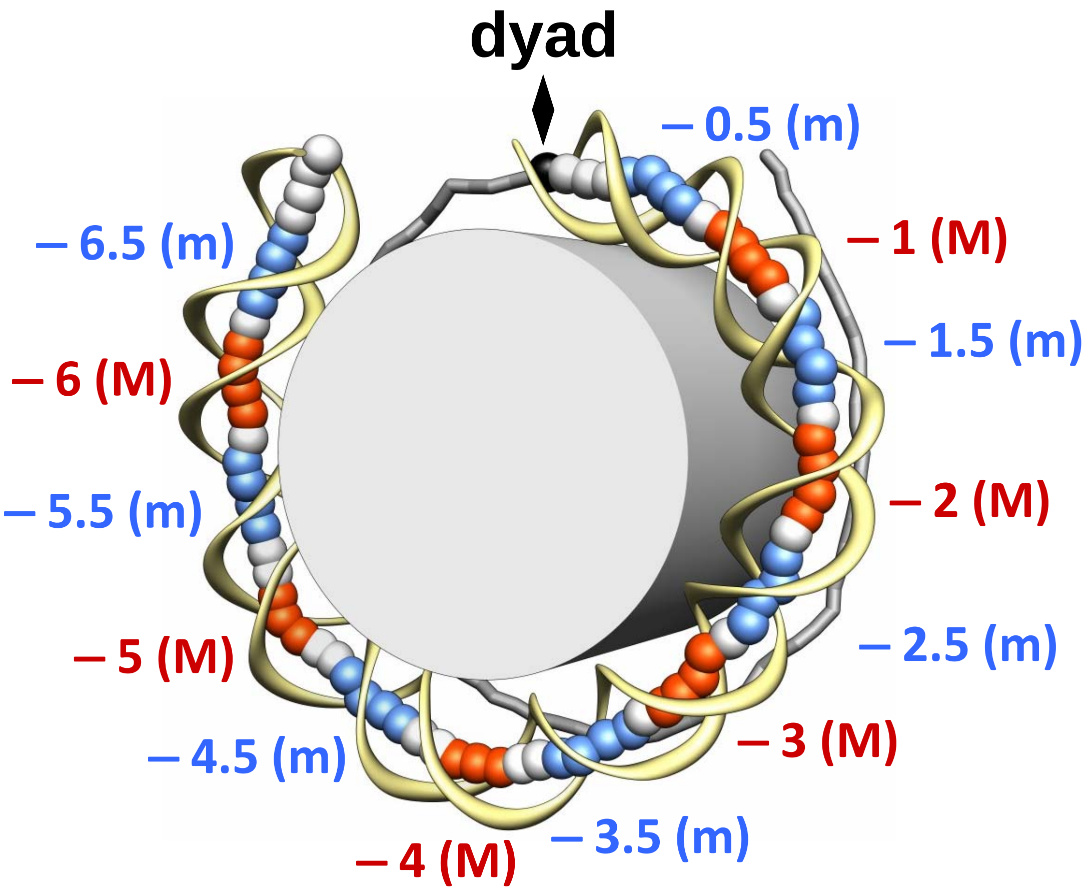

Figure S1

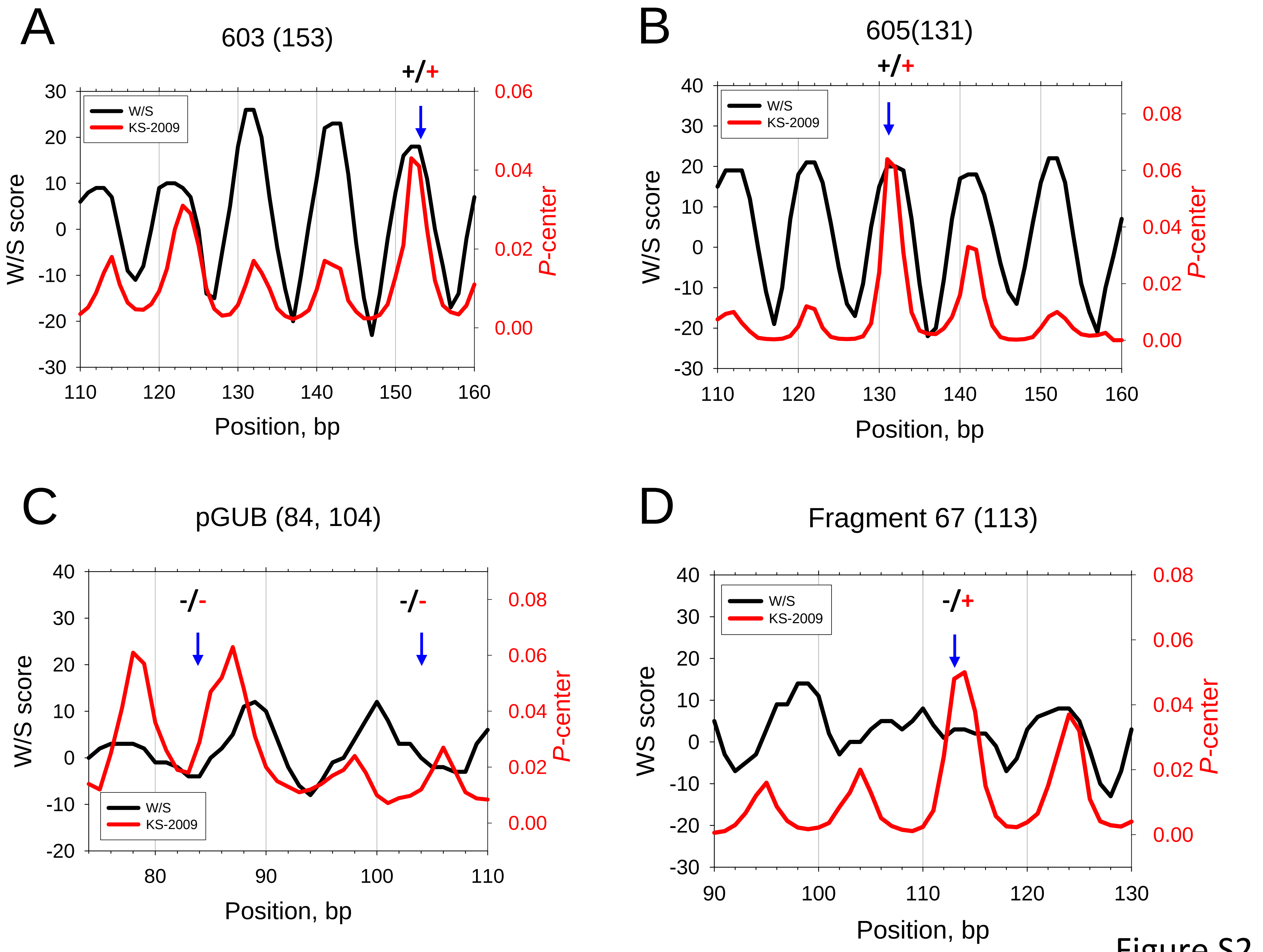
A
603 (153)
+/+
W/S
KS-2009
W/S score
P-center
Position, bp
B
605(131)
+/+
W/S
KS-2009
W/S score
P-center
Position, bp
C
pGUB (84, 104)
-/-
-/-
W/S
KS-2009
W/S score
P-center
Position, bp
D
Fragment 67 (113)
-/+
W/S
KS-2009
WS score
P-center
Position, bp

Figure S2

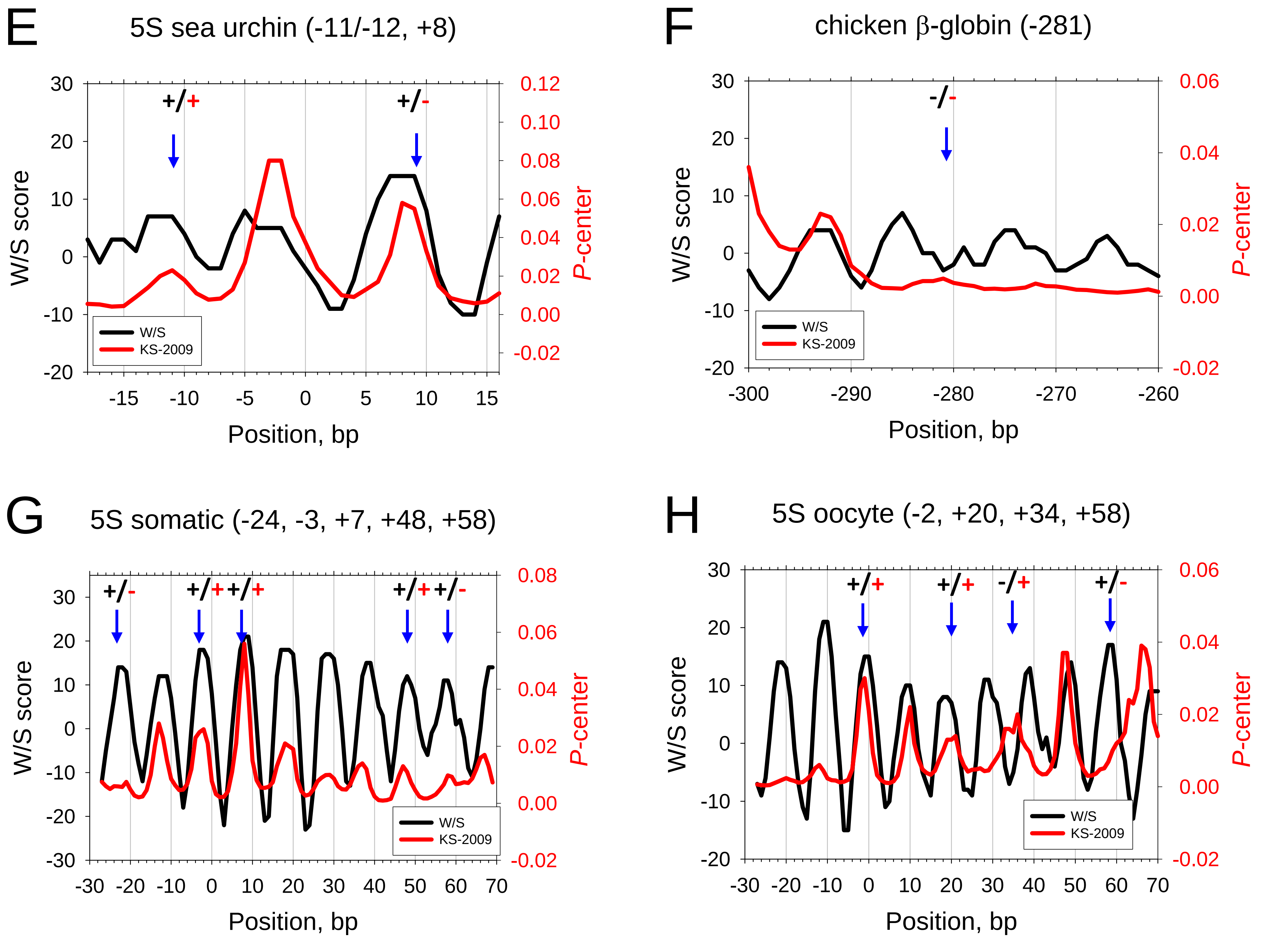
E
5S sea urchin (-11/-12, +8)
+/+
+/-
W/S score
P-center
W/S
KS-2009
Position, bp
F
chicken β-globin (-281)
-/-
W/S score
P-center
W/S
KS-2009
Position, bp
G
5S somatic (-24, -3, +7, +48, +58)
+/-
+/+
+/+
+/+
+/-
W/S score
P-center
W/S
KS-2009
Position, bp
H
5S oocyte (-2, +20, +34, +58)
+/+
+/+
-/+
+/-
W/S score
P-center
W/S
KS-2009
Position, bp

Figure S2

I

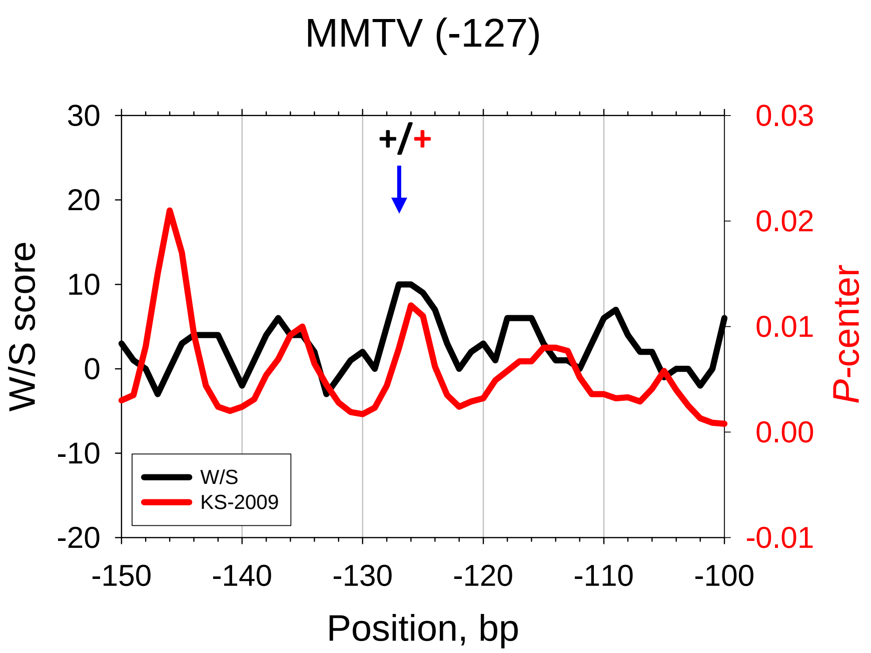
MMTV (-127)
+/+
W/S score
P-center
Position, bp
W/S
KS-2009
30
20
10
0
-10
-20
0.03
0.02
0.01
0.00
-0.01
-150
-140
-130
-120
-110
-100

J

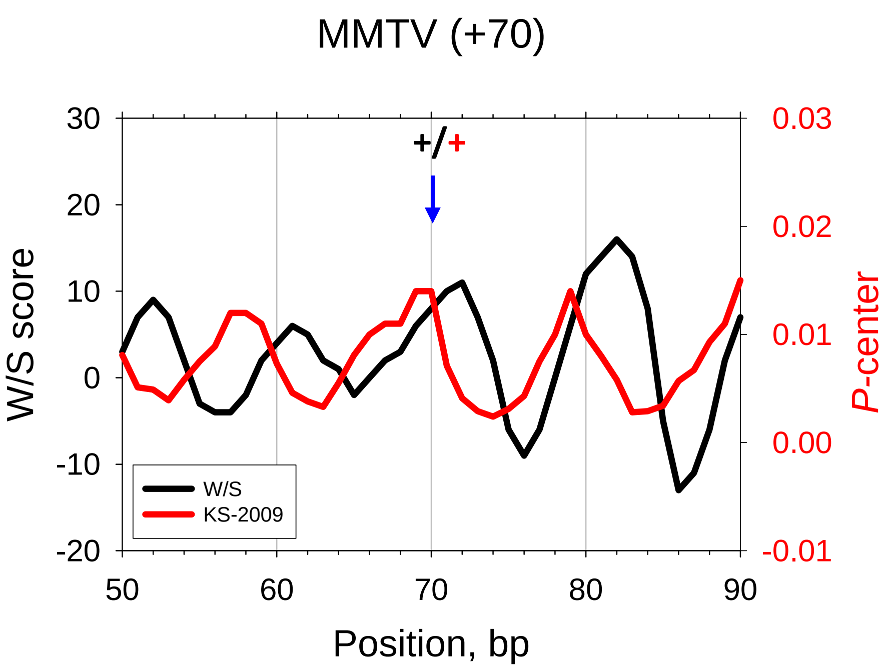
MMTV (+70)
+/+
W/S score
P-center
Position, bp
W/S
KS-2009
30
20
10
0
-10
-20
0.03
0.02
0.01
0.00
-0.01
50
60
70
80
90

Figure S2

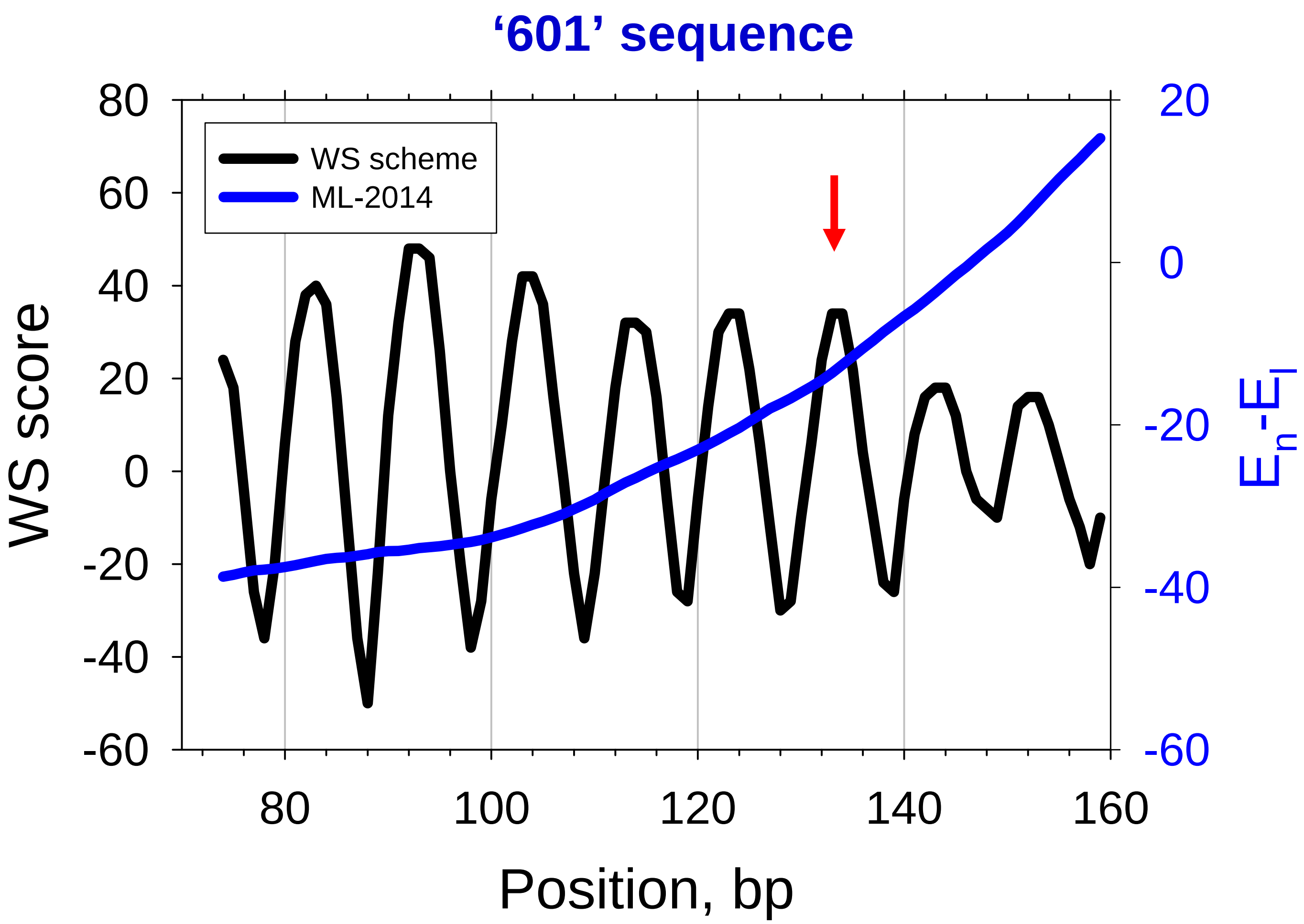

'601' sequence
WS scheme
ML-2014
WS score
E_n-E_l
Position, bp
80
60
40
20
0
-20
-40
-60
80
100
120
140
160
20
0
-20
-40
-60

Figure S3

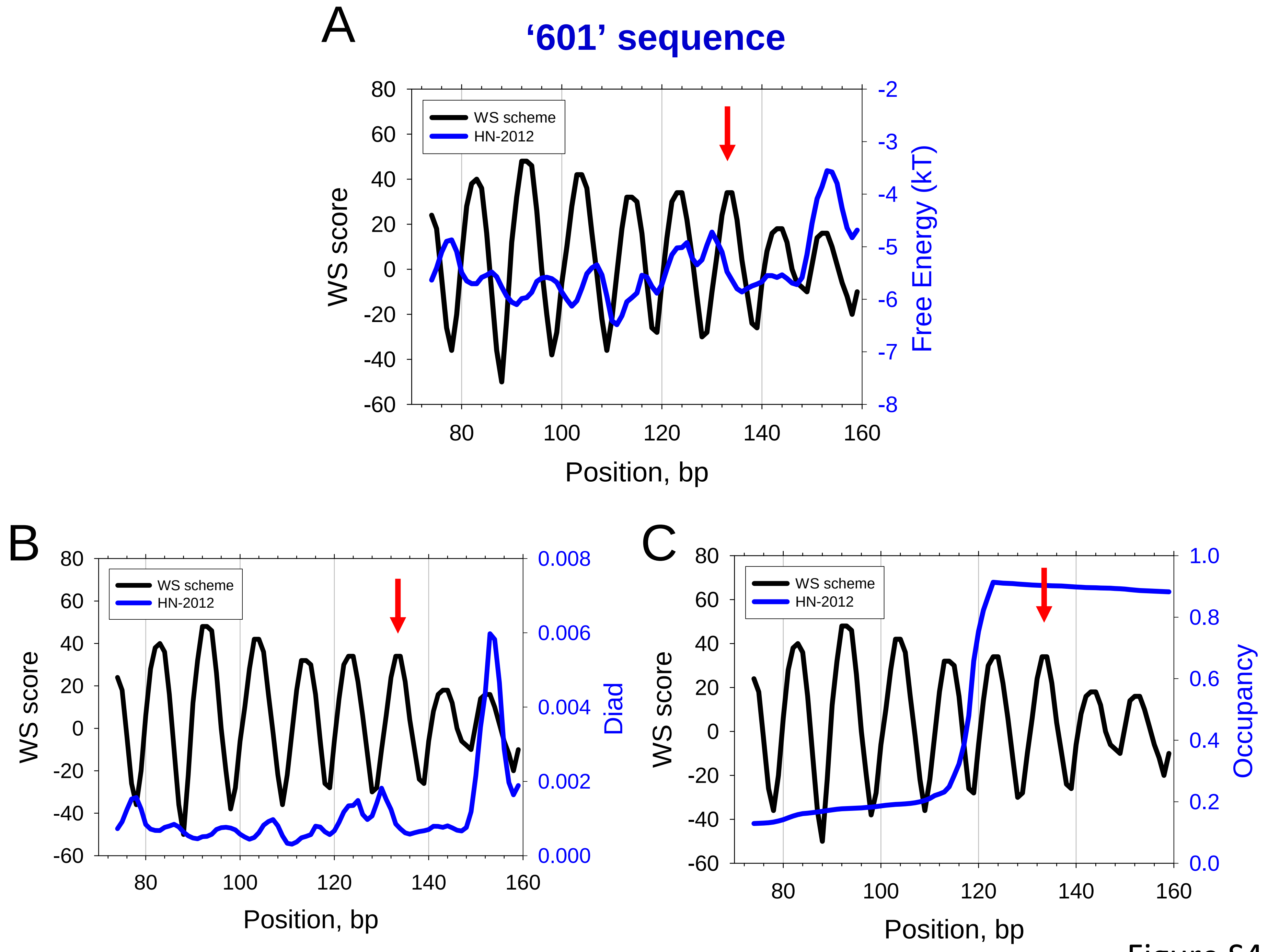
A
'601' sequence
WS scheme
HN-2012
WS score
Free Energy (kT)
Position, bp
80
60
40
20
0
-20
-40
-60
-2
-3
-4
-5
-6
-7
-8
100
120
140
160
B
Diad
0.008
0.006
0.004
0.002
0.000
C
Occupancy
1.0
0.8
0.6
0.4
0.2
0.0

Figure S4
